# Cost-effectiveness of eliminating hospital understaffing by nursing staff: a retrospective longitudinal study and economic evaluation

**DOI:** 10.1136/bmjqs-2024-018138

**Published:** 2025-04-29

**Authors:** Christina Saville, Jeremy Jones, Paul Meredith, Chiara Dall’Ora, Peter Griffiths

**Affiliations:** 1 University of Southampton, Southampton, UK; 2 National Institute for Health Research Applied Research Collaboration Wessex, Hampshire, UK

**Keywords:** Nurses, Health services research, Health policy, Cost-effectiveness, Patient Safety

## Abstract

**Background:**

Understaffing by nursing staff in hospitals is linked to patients coming to harm and dying unnecessarily. There is a vicious cycle whereby poor work conditions, including understaffing, can lead to nursing vacancies, which in turn leads to further understaffing. Is hospital investment in nursing staff, to eliminate understaffing on wards, cost-effective?

**Methods:**

This longitudinal observational study analysed data on 185 adult acute units in four hospital Trusts in England over a 5-year period. We modelled the association between a patient’s exposure to ward nurse understaffing (days where staffing was below the ward mean) over the first 5 days of stay and risk of death, risk of readmission and length of stay, using survival analysis and linear mixed models. We estimated the incremental cost-effectiveness of eliminating understaffing by registered nurses (RN) and nursing support (NS) staff, estimating net costs per quality-adjusted life year (QALY). We took a hospital cost perspective.

**Findings:**

Exposure to RN understaffing is associated with increased hazard of death (adjusted HR (aHR) 1.079, 95% CI 1.070 to 1.089), increased chance of readmission (aHR 1.010, 95% CI 1.005 to 1.016) and increased length of stay (ratio 1.687, 95% CI 1.666 to 1.707), while exposure to NS understaffing is associated with smaller increases in hazard of death (aHR 1.072, 95% CI 1.062 to 1.081) and length of stay (ratio 1.608, 95% CI 1.589 to 1.627) but reduced readmissions (aHR 0.994, 95% CI 0.988 to 0.999). Eliminating both RN and NS understaffing is estimated to cost £2778 per QALY (staff costs only), £2685 (including benefits of reduced staff sickness and readmissions) or save £4728 (including benefits of reduced lengths of stay). Using agency staff to eliminate understaffing is less cost-effective and would save fewer lives than using permanent members of staff. Targeting specific patient groups with improved staffing would save fewer lives and, in the scenarios tested, cost more per QALY than eliminating all understaffing.

**Interpretation:**

Rectifying understaffing on inpatient wards is crucial to reduce length of stay, readmissions and deaths. According to the National Institute for Health and Care Excellence £10 000 per QALY threshold, it is cost-effective to eliminate understaffing by nursing staff. This research points towards investing in RNs over NS staff and permanent over temporary workers. Targeting particular patient groups would benefit fewer patients and is less cost-effective.

**WHAT IS ALREADY KNOWN ON THIS TOPIC**

- In a literature review of 23 economic studies exploring the effect of variation in nurse staffing in acute hospital inpatient settings, all were judged to be at moderate or high risk of bias.
- Six studies found that increased nurse staffing levels were associated with improved outcomes and reduced or unchanged net costs, but most showed improved outcomes and increased costs.

**WHAT THIS STUDY ADDS**

- This study provides evidence of higher methodological quality that increasing nurse staffing is likely to be a cost-effective intervention.
- It suggests that eliminating understaffing on hospital wards by increasing registered nurse (RN) and nursing support worker staffing is likely to improve outcomes and increase costs, or save costs if reduced lengths of stay are accounted for.

**HOW THIS STUDY MIGHT AFFECT RESEARCH, PRACTICE OR POLICY**

- This study indicates the importance of prioritising investment in RNs employed on wards over nursing support worker staff, as well as showing there are no shortcuts to employing enough RNs as using temporary staff is more costly and less effective.

## Background

 Patients are coming to harm, and dying unnecessarily, due to hospital unit (ward) nurse understaffing.^1 2^ In some cases, this understaffing is driven by unfilled vacancies due to local or national shortages of nurses (in England, the current registered nurse (RN) vacancy rate stands at 10.6% or 43 339 vacancies^3^), but in other cases, it is the product of hospital decisions to limit the number of nurses employed. Since nursing forms the largest group of healthcare workers in England at approximately 60%,^4^ employing large numbers of RNs is clearly costly to hospitals. Thus, alternatives to employing RNs, such as investing in new roles with lower training requirements, are being pursued.^5^ Wards with insufficient nursing staff on a shift often rely on expensive agency staff to fill shortfalls.^6^ Alternative strategies include redeploying staff from other units or using in-house ‘bank’ or on-call staff, but this is difficult if all wards are running with low baseline staffing levels.^7^ In a survey of nurses, understaffing is one of the top reasons cited for leaving healthcare employment and hence contributing to a shortage of nurses,^8^ while burnout, which is associated with exposure to chronic understaffing,^9^ is another top reason for leaving.^8^

Much of the existing research in this area is cross-sectional and hence of limited use in determining cause and effect. A recent literature review of longitudinal studies of nurse staffing shows that higher RN staffing levels are associated with reduced risks of patient mortality, although the effect size remains uncertain.^1^ Evidence of the link between RN staffing and other patient outcomes such as readmissions and lengths of stay is less conclusive. Similarly, evidence for other nursing groups such as nursing support (NS) staff is less clear.

Researchers have previously attempted to quantify the value for money of increasing nurse staffing using a variety of economic study designs. However, according to a recent systematic review of 23 such studies, all studies suffered from moderate or high risk of bias.^10^ The main limitations were relying on staffing-outcome estimates from cross-sectional or single-site longitudinal studies, and mismatches between the patient group cared for by staff included and the patient group whose outcomes were measured. Overcoming these weaknesses would provide a more reliable estimate of cost-effectiveness.

This paper aims to answer the following research question: is investing in higher nurse staffing levels cost-effective? To address this, our objectives were (1) to estimate the longitudinal relationships between RN/NS staffing levels on acute adult inpatient wards (units) and mortality, readmissions and length of stay and (2) to estimate the incremental cost-effectiveness of eliminating understaffing.

## Methods

### Design and setting

This was a retrospective longitudinal study, part of a wider study estimating the costs and consequences of different workforce configurations in English acute hospitals.^11^ It made use of routinely collected data, linking daily ward-level nursing team roster data to individual patient records. The study protocol, including the health economic analysis plan, can be accessed at https://fundingawards.nihr.ac.uk/award/NIHR128056.

In England, the healthcare system (the National Health Service, NHS) is publicly funded and secondary care is provided in acute hospital Trusts which are groupings of one or more hospitals in the same area. The study data were provided by four English NHS hospital Trusts with diverse nurse staffing levels, sizes, teaching status and regions (with correspondingly diverse patient populations in terms of ethnicity mix and employment status/profession). Three Trusts provided acute inpatient services predominantly from single hospital sites, and the fourth provided inpatient services across four sites within one city.

### Data sources

Data covered April 2015 to March 2020, although discontinuities and transitions between roster systems meant that we were unable to access data for the entire period from all Trusts. The sample consisted of 626 313 patients staying in the four hospital Trusts. Patient data were extracted from electronic care records and consisted of patient demographics (gender and 5-year age bracket), method of admission, patient movements between wards and clinical International Classification of Diseases 10th Revision diagnosis codes.

Staffing data came from electronic roster systems. These data consisted of the worked shifts with details of pay band, post and fulfilment type (substantive, internal ‘bank’ or agency). There were two main nursing team roles: RNs (pay band 5 or higher) who have undertaken university undergraduate-level training and have a professional registration, and NS staff (pay band 2–4) who are largely unregulated.

### Inclusion/exclusion criteria

Patients were included if they stayed for at least one night in an acute adult inpatient area and were in the age bracket category of 15–19 years old or higher (age was supplied in 5-year age brackets for identity protection reasons). There was no restriction based on diagnosis. Staff shifts were included if they were worked by nursing staff, but student and non-clinical shifts were excluded.

### Outcomes

The primary outcome was death from all causes within 30 days of admission (both in or out of hospital). Secondary outcomes were length of stay and non-elective readmission to the same hospital Trust within 30 days of discharge (excluding admissions which ended with death). We did not have data on readmissions to other hospital Trusts. We also estimated costs of eliminating understaffing as cost per life saved and cost per quality-adjusted life year (QALY).

### Variables

The primary exposure variables of interest were RN and NS staffing levels, measured in hours per patient day. In this study, a day was a 24-hour period starting at 07:00, to correspond with the morning shift start time. To calculate patient days, we used records of patients being admitted, transferred and discharged. For each job group, hours per patient day were calculated per ward study day (worked staff hours/patient days).

As we had no consistent measure of the planned staffing, the ward mean (mean average worked hours per patient day for each ward) was used as a proxy, previously shown to be correlated.^12^ Where we saw changes in ward use or case mix for the ward over time, we divided the time series and calculated the mean staffing level for each subperiod separately. Days where staffing was below the ward mean were classified as understaffed. Thus, our analysis of understaffing is in relation to variation in each ward’s actual staffing levels, and those levels are determined by planned levels, recruitment policies and successes to recruit to those levels, and unplanned absences that could not be mitigated.

To adjust for individual variation in risk of death, we calculated the patients’ risk scores from their age, diagnostic group and comorbidities following the method used in the calculation of Summary Hospital-level Mortality Indicators (SHMI), but without aggregating to the hospital level. We used the SHMI specification version 1.30, issued on 30 April 2019,^13^ which is applicable to our study period.

### Statistical analyses

We used a mixed-effects (multilevel) modelling framework, with patients nested in wards, that is, ward-level variability in the outcomes was treated as a random effect. The ward variable was defined as the ward where the patient started the day (for the mortality and readmission models) and the discharge ward (for the length of stay model). We found that models with effects for both Trust and ward had negligible difference in Akaike information criterion (AIC, 0.4) and Bayesian information criterion (BIC, 1.2) from models with just ward effects, while providing almost exactly the same coefficients in our core models. We concluded that any Trust-level effects were properly accounted for by the ward random effect and so we omitted Trust from the models to reduce the computational intensity.

For mortality and readmission outcomes, we used a Cox survival analysis approach with staffing variables included as time-varying covariates.^14^ The assumption of proportional hazards was examined with graphs of scaled Schoenfeld residuals and was seen to be reasonable for the staffing measures. Because the effect of understaffing may accumulate over time, we modelled understaffing (yes or no) with cumulative time-dependent covariates in the survival models. For this, we constructed repeated observations (one per day) on each patient from the admission (ie, onset of risk) until death/readmission or censoring at 30 days. Thus, the exposure variable was the count of days that a patient was exposed to understaffing during the first (up to) 5 days of the hospital stay, accounting for most of the stay for most patients and the time when patients are likely to be most acutely ill; this avoids problems with bias due to patients staying longer having more chance of being exposed to understaffing.^15 16^

For length of stay, we used a generalised linear regression model with a gamma distribution, as length of stay is not normally distributed. In the length of stay model, the exposure variable was the proportion of understaffed days during the first 5 days of the hospital stay. This is a different exposure variable from that used in the mortality and readmission models because we are not using a survival modelling framework so do not have repeated observations.

We compared model fits, where appropriate, using the AIC and BIC. We conducted subgroup analyses for highly acute and less acute patients, as measured by the National Early Warning Score on admission (scoring ≥5 or <5 respectively), and patients in older people wards (at least 75% of people aged at least 75 years old). We conducted sensitivity analyses including using different thresholds for understaffing, with time of year for seasonal effects and a weekend variable as a proxy for lower staffing from non-nursing staff groups.

### Economic analyses

We estimated the incremental cost-effectiveness of eliminating understaffing by RN and NS staff. For this, we estimated the costs and consequences of moving from the observed staffing shortfall averaged over the study period to the planned staffing. The quantity of staff hours required to eliminate understaffing was estimated from the count of patient days in the study, the proportion of understaffed days and the average shortfall (in RN and NS hours per patient day). We took a hospital cost perspective (since budgets are managed at this level in the NHS) so included costs relating to the index admission and readmissions shortly after (within 30 days of discharge). Costs of estimated change in length of stay associated with staffing change were valued using the NHS reference costs/tariff for excess bed days.^17^ Costs of initial admissions/readmissions were based on the distribution of Health Resource Group codes^18^ in the original cohort/for observed readmissions together with the national reference cost. Costs of staff were based on nationally representative unit costs for substantive staff^19^ (online supplemental table 1).

Using statistical models for mortality, readmissions, length of stay (all reported in this paper) and sickness absence,^20^ we extrapolated the effects of avoiding understaffing. For mortality and readmissions, we assumed that the HR approximated to a risk ratio, which is generally valid in the short run and when event probabilities are small.^21 22^ We applied the risks over the average patient exposure to understaffing during the first 5 days. The combined (multiplicative) effects were applied to the observed mortality rate. The difference between the observed and modelled death rates was applied to the total number of deaths observed to estimate the number of deaths averted by avoiding staffing shortfalls.

We used the discounted and quality-adjusted life expectancy approach to estimate QALYs associated with deaths avoided,^23^ thus taking a lifetime perspective on outcomes to fully capture the effect. This approach combines age/sex-specific life expectancy estimates with quality-of-life valuations on the utility scale (where 0 means death and 1 means perfect health). We applied a discount rate of 3.5% per year, so that years of life in the distant future have less value than current years.^24^ We used Office for National Statistics life tables to estimate age and sex-specific life expectancy.^25^ Since our data did not have precise patient ages (for confidentiality and identifiability reasons), we calculated a sex-specific weighted average mortality rate for each age band. This was based on the population distribution in each age within each age band using the LifeTable() function from the R library MortalityLaws.^26^ We estimated QALY expectations for the patients who died (derived from the Health Survey for England) and assumed that if those patients were to survive, the age group/sex-specific life expectancy would be the same as that of a similar individual with an existing condition in the general population^27^ (online supplemental table 2).

We conducted a range of sensitivity analyses and subgroup analyses (online supplemental material page 3). We varied economic parameters and effect sizes and tested scenarios where understaffing was only partially eliminated. We considered the use of temporary staff since they are often used to make up shortfalls. We accounted for their reduced effectiveness; our previously published regression modelling on this dataset^28^ showed that while agency staff save lives compared with leaving shifts understaffed, they are less effective than permanent staff at reducing the risk of death (62% fewer lives saved). In subgroup analyses, we explored the cost-effectiveness of targeting particular patient groups as this could focus rectifying shortfalls where most needed.

## Results

There were 185 adult acute units and 626 313 patient admissions treated at four hospital Trusts in our analysis dataset, with patients spending an average of 3.6 days in hospital. Over the first 5 days of stay, on average, patients were provided with 5.3 hours of care every day from RNs and 2.9 hours of care from NS staff. Further details of patient characteristics and staffing are presented in online supplemental tables 3 and 4.

Unadjusted associations between understaffing (staffing below the ward mean within the first 5 days) and mortality, readmission and length of stay are reported in online supplemental table 5. Patients exposed to understaffing by RNs were more likely to die (5% vs 4%), be readmitted (15% vs 14%) and stay in hospital longer (8 days vs 5 days), with similar figures for NS understaffing. For those exposed to understaffing, they were exposed to an average 1.15-hour shortfall in the first 5 days, while those not exposed to understaffing were exposed, on average, to 3.36 care hours above the ward mean.

In the multivariable models, each day a patient was exposed to understaffing by RNs (staffing below the ward mean) in the first 5 days of their stay, the hazard of death increased by 8% (adjusted HR (aHR) 1.079, 95% CI 1.070 to 1.089) and the hazard of readmission increased by 1% (aHR 1.010, 95% CI 1.005 to 1.016) (table 1). When all days of a patient stay within 5 days of admission were understaffed, the length of stay was increased by 69%. Days of understaffing by NS staff were also associated with increases, although slightly smaller, in hazard of death (aHR 1.072, 95% CI 1.062 to 1.081) and length of stay (ratio 1.608, 95% CI 1.589 to 1.627). However, for readmissions, the effect was in the opposite direction; for each day of understaffing by NS staff, the hazard of readmission decreased by 0.6% (aHR 0.994, 95% CI 0.988 to 0.999).

**Table 1:** Association between days of understaffing and outcomes, adjusted for risk

Term	Mortality				Readmission				Length of stay			
	aHR	P value	LCL	UCL	aHR	P value	LCL	UCL	Ratio*	P value	LCL	UCL
Mortality risk score	1.063	0.000	1.062	1.064	1.006	0.000	1.005	1.007	1.032	0.000	1.031	1.032
RN understaffing†	1.079	0.000	1.070	1.089	1.010	0.000	1.005	1.016	1.687	0.000	1.666	1.707
NS understaffing†	1.072	0.000	1.062	1.081	0.994	0.020	0.988	0.999	1.608	0.000	1.589	1.627
Ward random effect (SD)	1.205				0.657				0.902			
	**AIC**	**BIC**			**AIC**	**BIC**			**AIC**	**BIC**		
Model fit	788 843	790 453			2 424 881	2 426 790			7 025 587	7 025 655		

* Multiplier for length of stay for a unit change in the independent variable.

† Understaffing is defined as staffing below the ward mean within the first 5 days. For mortality and readmission, the exposure is days of understaffing. For length of stay, the exposure is the proportion of days in the first 5 days of patient stay with understaffing.

aHR, adjusted HR; AIC, Akaike information criterion; BIC, Bayesian information criterion; LCL, lower 95% confidence limit; NS, nursing support; RN, registered nurse; UCL, upper 95% confidence limit.

### Patient subgroup and sensitivity analyses

Associations were similar for less acute (National Early Warning Score <5) patients as for all eligible patients (see table 2). There was a similar pattern of results for staffing on wards for older people, although effect sizes were smaller and readmissions were not associated with RN understaffing. For highly acute patients (National Early Warning Score 5+), RN understaffing was associated with increased length of stay and increased deaths, while NS understaffing was associated with increased length of stay but reduced mortality and readmission. The estimated effect of low staffing did not alter when including time of year for seasonal effect and a weekend variable as a proxy for other staff groups. Sensitivity analyses with different thresholds for understaffing showed similar results, apart from RNs where using thresholds higher than the mean showed greater effects, likely to lead to improved cost-effectiveness (online supplemental figure 1).

**Table 2:** Association between days of understaffing and outcomes for patient and ward subgroups

Subgroup	Mortality				Readmission				Length of stay			
Less acute (National Early Warning Score <5) patients	aHR	P value	LCL	UCL	aHR	P value	LCL	UCL	Ratio*	P value	LCL	UCL
Mortality risk score	1.064	0.000	1.063	1.065	1.006	0.000	1.005	1.006	1.030	0.000	1.029	1.030
RN understaffing†	1.094	0.000	1.081	1.106	1.011	0.000	1.005	1.018	1.625	0.000	1.602	1.649
NS understaffing†	1.087	0.000	1.075	1.099	0.995	0.110	0.989	1.001	1.567	0.000	1.545	1.590
Ward random effect (SD)	0.961				0.612				0.920			
	**AIC**	**BIC**			**AIC**	**BIC**			**AIC**	**BIC**		
Model fit	497 531	498 792			1 769 632	1 771 200			4 937 519	4 937 585		

Highly acute (National Early Warning Score 5+) patients	aHR	P value	LCL	UCL	aHR	P value	LCL	UCL	Ratio*	P value	LCL	UCL
Mortality risk score	1.050	0.000	1.048	1.052	1.001	0.514	0.998	1.004	1.032	0.000	1.031	1.032
RN understaffing†	1.036	0.001	1.014	1.059	0.988	0.244	0.968	1.008	1.664	0.000	1.644	1.684
NS understaffing†	0.978	0.033	0.958	0.998	0.974	0.008	0.955	0.993	1.605	0.000	1.586	1.624
Ward random effect (SD)	0.645				0.391				0.916			
	**AIC**	**BIC**			**AIC**	**BIC**			**AIC**	**BIC**		
Model fit	107 720	108 462			103 017	103 669			6 922 804	6 922 871		

Older people’s wards‡	aHR	P value	LCL	UCL	aHR	P value	LCL	UCL	Ratio*	P value	LCL	UCL
Mortality risk score	1.053	0.000	1.052	1.054	0.993	0.000	0.992	0.994	1.019	0.000	1.018	1.019
RN understaffing†	1.048	0.000	1.037	1.060	0.999	0.798	0.990	1.007	1.422	0.000	1.396	1.449
NS understaffing†	1.047	0.000	1.036	1.059	0.986	0.001	0.978	0.995	1.447	0.000	1.421	1.474
Ward random effect (SD)	0.569				0.481				0.695			
	**AIC**	**BIC**			**AIC**	**BIC**			**AIC**	**BIC**		
Model fit	481 098	482 323			805 751	807 111			2 415 199	2 415 260		

* Multiplier for length of stay for a unit change in the independent variable.

† Understaffing is defined as staffing below the ward mean within the first 5 days. For mortality and readmission, the exposure is days of understaffing. For length of stay, the exposure is the proportion of days in the first 5 days of patient stay with understaffing.

‡ Over 75% of patients over 75 years old.

aHR, adjusted HR; AIC, Akaike information criterion; BIC, Bayesian information criterion; LCL, lower 95% confidence limit; NS, nursing support; RN, registered nurse; UCL, upper 95% confidence limit.

### Economic results

Based on the weighted average Health Resource Group codes cost, the estimated total cost of providing care for the 626 313 adult inpatient admissions in our dataset was £2 613 385 125, or £4173 per admission. The mean estimated discounted and quality-adjusted life expectancy among patients who died was 6.82. This was the figure we used in our base case scenario to reflect the quality-adjusted life expectancy of any lives saved from reduced understaffing, equivalent to 6.82 years in full health gained per life saved from reduced understaffing.

We estimated that eliminating RN and NS understaffing would cost an additional £197 per patient admission, avoiding 6527 of 31 885 deaths over the study period and gaining 44 483 QALYs (table 3). This equates to an additional staff cost of £2778 per QALY. However, if we account for the value of reduced staff sickness absences and averted readmission stays due to the higher staffing levels, the net cost per QALY is reduced to £2685. Accounting for reduced lengths of stay as well gives a net cost reduction of £4728 per QALY, that is, an overall cost saving from increasing staffing levels.

**Table 3:** Costs, consequences and cost-effectiveness of eliminating understaffing (base case and sensitivity analysis)

Assumptions	Lives saved	QALY gains	Additional staff cost per admission	Value of reduced sickness absence (% of staff costs)	Value of averted readmissions (% of staff costs)	Value of reduced stays (% of staff costs)	Additional staff cost per life saved	Additional staff cost per QALY	Net additional cost per QALY (sickness costed)*	Net additional cost per QALY (sickness and readmissions costed)†	Net additional cost per QALY (sickness, readmissions and LOS costed)‡
Base case	6527	44 483	£197	1.4	2.0	267	£18 930	£2778	£2739	£2685	−£4728
Sensitivities											
Lower discount rate (3%)	6527	46 183	£197	1.4	2.0	267	£18 930	£2675	£2638	£2586	−£4554
Higher discount rate (5%)	6527	40 097	£197	1.4	2.0	267	£18 930	£3081	£3038	£2978	−£5245
High-risk populations saved (over 80)	6527	22 605	£197	1.4	2.0	267	£18 930	£5466	£5389	£5283	−£9304
LCL for all consequences	5746	39 158	£197	0.2	−1.7	261	£21 504	£3155	£3150	£3203	−£5041
UCL for all consequences	7303	49 773	£197	3.4	5.6	272	£16 918	£2482	£2399	£2261	−£4503
Lower value for unit costs	6527	44 483	£181	1.4	0.8	267	£17 363	£2548	£2513	£2492	−£4317
Higher value for unit costs	6527	44 483	£227	1.4	2.0	265	£21 788	£3197	£3153	£3088	−£5395
Optimistic cost assumptions§	6527	44 483	£181	1.4	2.5	333	£17 363	£2548	£2513	£2448	−£6035
Pessimistic cost assumptions¶	6527	44 483	£227	1.4	0.7	213	£21 788	£3197	£3153	£3132	−£3677

* Costs of avoided sickness absence removed.

† Costs of avoided sickness absence and readmissions removed.

‡ Costs of avoided sickness absence, readmissions and days of stay removed.

§ Lower limit for staffing unit costs, upper limit for readmission/length of stay unit costs (see Supplementary Table 1online supplemental table 1 for costs).

¶ Upper limit for staffing unit costs, lower limit for readmission/length of stay unit costs (see Supplementary Table 1online supplemental table 1 for costs).

LCL, lower 95% confidence limit; LOS, length of stay; QALY, quality-adjusted life year; UCL, upper 95% confidence limit.

The sensitivity analysis with the largest impact on results was assuming a lower quality-adjusted life expectancy for the lives saved. If it was assumed that the expectancy was lower than in the base case (mean expectancy), matching that of the (approximately) 50% of the cohort of deaths who were 80 years old or older, the staff cost per QALY was 97% higher (£5466). Varying discount rates and cost assumptions led to relatively small changes in staff cost per QALY (at most a 15% change).

Eliminating understaffing for particular patient groups would save fewer lives and cost more per QALY than eliminating understaffing for all, although since these are different populations, it is hard to compare estimates directly (table 4). We estimated the staff cost of targeting the most acute patients would be 254% more per QALY gained, the least acute 38% more and older people’s wards 311% more. Avoiding RN understaffing only would cost less per admission than avoiding all understaffing, but cost 27% more per QALY due to the reduced benefits.

**Table 4:** Costs, consequences and cost-effectiveness of eliminating low staffing for patient/ward subgroups and of partial reductions in understaffing

Scenario	Lives saved	QALY gains	Additional staff cost per admission	Additional staff cost per life saved	Additional staff cost per QALY
Highly acute (National Early Warning Score 5+) patients	141	926	£311	£64 786	£9848
Less acute (National Early Warning Score <5) patients	4902	32 577	£285	£25 388	£3820
Care of older people ward subgroup	384	2617	£427	£77 806	£11 416
Remove 50% understaffing	3356	22 875	£99	£18 405	£2701
Remove RN understaffing only	3627	24 717	£139	£24 071	£3532

QALY, quality-adjusted life year; RN, registered nurse.

We estimated the cost-effectiveness of eliminating understaffing using agency rather than permanent staff (table 5). For both RN and NS staff, the mean staffing shortfall was approximately 17% and so we used this figure to estimate the proportion of agency staff that would be needed. If agency staff are assumed to be as effective as permanent staff (same number of lives saved), then staff costs per QALY ranged from £2778 (if agency staff cost the same as permanent, equivalent to base case) to £5555 (if agency staff cost twice as much). Taking account of the reduced effectiveness of agency staff, and under assumptions that agency staff cost between the same and double what permanent staff are paid, the staff costs per QALY ranged from £7320 to £14 639 per QALY, respectively.

**Table 5:** Costs, consequences and cost-effectiveness of eliminating low staffing with agency staff (base case and sensitivity analysis)

Assumptions	Lives saved	QALY gains	Additional staff cost per admission	Additional staff cost per life saved	Additional staff cost per QALY	Net additional cost per QALY (sickness costed)*	Net additional cost per QALY (sickness and readmissions costed)†	Net additional cost per QALY (sickness, readmissions and LOS costed)‡
Agency staff cost the same as substantive staff, reduced effectiveness§	2477	16 879	£197	£49 887	£7320	£7218	£7075	−£12 460
Higher agency cost (125% of substantive staff cost)	6527	44 483	£247	£23 663	£3472	£3424	£3369	−£4043
Higher agency cost (125% of substantive staff cost), reduced effectiveness§	2477	16 879	£247	£62 358	£9150	£9022	£8879	−£10 656
Higher agency cost (150% of substantive staff cost)	6527	44 483	£296	£28 395	£4166	£4108	£4054	−£3359
Higher agency cost (150% of substantive staff cost), reduced effectiveness§	2477	16 879	£296	£74 830	£10 980	£10 826	£10 684	−£8851
Higher agency cost (200% of substantive staff cost)	6527	44 483	£395	£37 860	£5555	£5478	£5423	−£1989
Higher agency cost (200% of substantive staff cost), reduced effectiveness§	2477	16 879	£395	£99 773	£14 639	£14 435	£14 292	−£5243

* Costs of avoided sickness absence removed.

† Costs of avoided sickness absence and readmissions removed.

‡ Costs of avoided sickness absence, readmissions and days of stay removed.

§ Mortality effect adjusted for use of agency.

LOS, length of stay; QALY, quality-adjusted life year.

## Discussion

Our longitudinal economic study of four hospital Trusts has revealed four key findings. First, understaffing by RNs is associated with higher risks of patients dying, being readmitted and longer stays in hospital, while understaffing by NS staff is also associated with increased risks of dying and longer stays (by a slightly smaller amount), but reduced risks of readmission. Second, eliminating understaffing by nursing staff is estimated to cost £2778 per QALY, and while accounting for readmissions and staff sickness makes little difference to this estimate, if length of stay changes are accounted for, eliminating understaffing appears to save money. Third, using agency staff to eliminate understaffing appears less cost-effective and saves fewer lives than using permanent members of staff. Finally, targeting particular patient groups with improved staffing has fewer benefits, and for the groups we tested has a higher cost per QALY.

Our approach differs from much previous research because we use a threshold and do not assume a linear dose–response relationship.^1^ The most significant innovation is that we have explored for the first time the cost-effectiveness of targeting measures to avoid low staffing on specific patient groups, allowing decision-makers to compare cost-effectiveness between different strategies.

The findings give no indication that it makes rational economic sense to target efforts to rectify low staffing only on the most acute patients. Not only is this logistically difficult for patients whose acuity is emergent (occurring while on a general ward), it also gives much less benefit at a considerably higher cost per unit improvement in outcome. Steps to address low staffing for the general (lower acuity) population are likely to benefit high-acuity patients as well, in so far as they are in the same units, whereas the opposite is unlikely to occur if interventions are targeted on high-acuity patients in high-acuity units. A potential reason for this seemingly counterintuitive finding is that the sickest patients on admission may have a higher intrinsic risk and so less potential to benefit from treatment and early intervention for further deterioration, compared with those whose complications emerge during their stay.

Our study overcomes some of the main problems with bias in previous studies because it is a longitudinal multisite study and the estimates of staffing-outcome associations directly link patients to staffing at a day level, on the unit where patients are staying. We included a range of costs and took a long-term perspective by estimating QALYs associated with deaths avoided.

However, our study has limitations. Data came exclusively from hospitals in the English NHS, so results may differ for other settings and particularly where healthcare costs and funding mechanisms differ. This is an observational study and so associations used to estimate causal relationships for estimates of cost-effectiveness could be biased. Understaffing was judged relative to ward norms rather than a validated assessment of staffing need, although previous research in a similar setting showed these are highly correlated.^14^ There is uncertainty in the estimates of costs and consequences, although the presented scenarios indicate the likely range of results based on data from multiple sites. We did not have data on all the consequences of eliminating understaffing, and so our cost-effectiveness estimates are likely to be underestimates. In particular, our analyses did not account for prevented/reduced burnout or reduced turnover of nurses, which are costly problems to hospitals and health systems. Although we adjusted nominal life expectancies for age and sex, we did not adjust for diagnosis and comorbidities/frailty which could lead to an underestimate of costs per QALY.

Previous patient-level longitudinal studies have found higher RN staffing levels to be associated with reduced patient mortality, as in this study, but the evidence for NS staffing levels is inconsistent^4^; we found effects for NS workers in the same direction as for RNs but smaller. The only longitudinal study on readmissions that we are aware of focused on patients over the age of 75 who received a cognitive screening and found exposure to additional RN hours associated with reduced risk of readmission (although non-significant),^29^ which corresponds with the relationship we found for all patients. Our review of economic studies found a range of cost-effectiveness estimates from mostly cross-sectional evidence^9^; our estimate of staff cost per life saved is broadly comparable with several previous estimates and is based on longitudinal evidence. When accounting for the impact of staffing on readmissions and length of stay we found a net reduction in costs, consistent with findings from McHugh’s modelling of the effects of increased RN staffing in Australia.^30^

In the UK, since health services are nationally funded, there is a need to balance spending across services in a way that is fair and provides the safest and highest quality care. The National Institute for Health and Care Excellence identified £10 000 per QALY ($15 572 is the 2021 US$ equivalent) as representing ‘exceptional value for money’,^31^ meaning that a drug could be fast-tracked for availability in the NHS, and the majority of our estimates are below this. When considering alternative policy strategies, this study indicates the importance of prioritising investment in RNs employed on wards over support staff, as well as showing there are no shortcuts to employing enough RNs as using temporary staff is more costly and less effective.

To see if results translate to other settings, we recommend similar cost-effectiveness studies with longitudinal multisite designs are carried out in other countries. Future research should also address how to predict demand and absences to support rostering sufficient staff and reduce reliance on temporary staff.

## Supplementary material

10.1136/bmjqs-2024-018138 online supplemental file 1

## Data Availability

No data are available.
