# Supplementary material for: Cost-effectiveness of eliminating hospital understaffing by nursing staff: a retrospective longitudinal study and economic evaluation
Source: BMJ Qual Saf. 2025 Apr 29;35(1):e018138. doi: 10.1136/bmjqs-2024-018138 (PMC12772556; doi:10.1136/bmjqs-2024-018138)
Supplement: online supplemental file 1 [file bmjqs-35-1-s001.pdf]

Supplementary Material - Table of Contents

Methodological Tables 2
Details of economic sensitivity analyses and subgroup analyses 3
Descriptive statistics 4
Univariable analysis results 5
Sensitivity analysis: Alternative understaffing thresholds 6

Methodological Tables

Table 1 - Unit costs used in the analysis

Variable	Currency	Unit cost Base case (£)	Low (£)	High (£)
**Staffing costs[a]**				
Registered Nurse Band 5	Per hour of staff time	43		
Registered Nurse Band 6		53		
Assumed weighted average cost of replacement staff		45.35	42.70	53.31
Registered Nurse Band 7		64		
Nursing Assistants		33.45	28.72	36.52
**Hospital admission costs**				
Readmission[b]	Cost per readmission	2,906.50	1,118.40	3,468.43
Hospital stay[c]	Cost per day (excess bed day)	270	248	309

Sources:
a.Unit Costs of Health and Social Care (2022) (1)
b. National (NHS) reference costs for hospital stay(2)
c. Costs of readmissions were based on the distribution of Health Resource Group codes(3)

Assumptions:

- Base case Registered Nurse cost is based on mean salary and on-costs for band 5 and 6 (weighted 75% band 5 and 25% band 6 based on observed distribution of senior / junior staff). Low estimate is based on mean band 5 salary and on-costs and high estimate is based on mean band 6 salary with on-costs.
- Base case Nursing Assistant cost is based on mean salary and on-costs for band 4. Low estimate is based on the entry point for Band 2 and high estimate is based on max annual salary for band 4 with on-costs.
- Readmission cost was estimated as the weighted average of HRG costs for readmissions observed in study dataset. Low value is the 25th percentile of this distribution and high value is the 75th percentile.
- Hospital cost per day (for reduction in length of stay) was estimated as the weighted average of excess bed costs reported in National Tariff for 21/22 (weighted by activity reported to generate costs). Low value is the 25th percentile of this distribution and high value is the 75th percentile.

Table 2 - Abridged life table life expectancy estimates, discounted life expectancy, quality of life weights and DANQALE

Age group	Life expectancy		Discounted life expectancy		At least one condition	Discounted Quality Adjusted Life Expectancy	
	Female	Male	Female	Male		Female	Male
**0**	82.66	78.93	27.85	27.61	0.855	23.8	23.6
**1 - 4**	81.94	78.26	27.81	27.57	0.855	23.8	23.6
**5 - 9**	77.98	74.31	27.55	27.28	0.855	23.6	23.3
**10 - 14**	73.01	69.33	27.17	26.85	0.855	23.2	23.0
**15 - 19**	68.03	64.36	26.72	26.34	0.855	22.8	22.5
**20 - 24**	63.09	59.46	26.20	25.75	0.855	22.4	22.0
**25 - 29**	58.15	54.61	25.57	25.05	0.855	21.9	21.4
**30 - 34**	53.23	49.78	24.83	24.24	0.814	20.2	19.7
**35 - 39**	48.35	44.99	23.97	23.28	0.809	19.4	18.8
**40 - 44**	43.52	40.26	22.95	22.17	0.763	17.5	16.9
**45 - 49**	38.75	35.62	21.77	20.89	0.757	16.5	15.8
**50 - 54**	34.08	31.09	20.41	19.42	0.739	15.1	14.4
**55 - 59**	29.49	26.69	18.85	17.76	0.740	13.9	13.1
**60 - 64**	25.03	22.44	17.07	15.90	0.726	12.4	11.5
**65 - 69**	20.75	18.41	15.09	13.87	0.741	11.2	10.3
**70 - 74**	16.67	14.67	12.90	11.72	0.718	9.3	8.4
**75 - 79**	12.83	11.19	10.55	9.45	0.697	7.4	6.6
**80 - 84**	9.40	8.18	8.17	7.25	0.638	5.2	4.6

**85 - 89**	6.48	5.68	5.90	5.25	0.595	3.5	3.1
**90 - 94**	4.16	3.78	3.94	3.60	0.595	2.3	2.1
**95 +**	2.13	2.06	2.09	2.02	0.595	1.2	1.2

Details of economic sensitivity analyses and subgroup analyses

We conducted sensitivity analyses including assuming a higher mortality expectation (hence shorter life expectancy) and varying the discount rate and effect sizes. We conducted subgroup analyses for highly acute patients and less acute patients, and patients in older people wards. We also ran scenarios where only RN understaffing was eliminated and where only 50% of understaffing was eliminated and investigated the cost-effectiveness of eliminating understaffing using agency staff. We focused on agency staff rather than bank staff because these are potentially more expensive, and the adverse effects were either similar or worse. As prices paid for agency staff can be highly variable, we estimated various proportional increases compared to the cost of permanent staff. In the base case we assumed the same effectiveness as for permanent staff but in sensitivity analyses, we tested an assumption of reduced effectiveness at saving lives based on regression models.(4)

Descriptive statistics

Table 3 Adult inpatient admissions

	All		Alive**		Dead**	
	n	(%)	n	(%	n	(%)
**Total N**	626313	(100.0%)	594428	(94.9%)	31885	(5.1%)
**Female**	348464	(55.6%)	333411	(56.1%)	15053	(47.2%)
**Emergency admission**	502717	(80.3%)	471895	(79.4%)	30822	(96.7%)
**Age group** 15-19	13699	(2.2%)	13665	(2.3%)	34	(0.1%)
20-24	25980	(4.1%)	25927	(4.4%)	53	(0.2%)
25-29	33779	(5.4%)	33702	(5.7%)	77	(0.2%)
30-34	36544	(5.8%)	36413	(6.1%)	131	(0.4%)
35-39	29740	(4.7%)	29543	(5.0%)	197	(0.6%)
40-44	23420	(3.7%)	23131	(3.9%)	289	(0.9%)
45-49	28092	(4.5%)	27589	(4.6%)	503	(1.6%)
50-54	34560	(5.5%)	33756	(5.7%)	804	(2.5%)
55-59	38843	(6.2%)	37595	(6.3%)	1248	(3.9%)
60-64	42138	(6.7%)	40401	(6.8%)	1737	(5.4%)
**65-69***	49617	(7.9%)	47071	(7.9%)	2546	(8.0%)
70-74	60212	(9.6%)	56532	(9.5%)	3680	(11.5%)
75-79	59095	(9.4%)	54974	(9.2%)	4121	(12.9%)
80-84	60436	(9.6%)	55156	(9.3%)	5280	(16.6%)
85-89	51579	(8.2%)	45983	(7.7%)	5596	(17.6%)
90-120	38579	(6.2%)	32990	(5.5%)	5589	(17.5%)
**Charlson Comorbidity Index**						
[0]	245515	(39.2%)	242986	(40.9%)	2529	(7.9%)
[1-5]	100037	(16.0%)	97842	(16.5%)	2195	(6.9%)
[>5]	279415	(44.6%)	252275	(42.4%)	27140	(85.1%)
**Length of stay**						
Mean (SD)	7.85	(13.88)	7.79	(14.15)	9.0	(7.00)
Median (IQR)	3.63	(1.77 to 8.28)	3.46	(1.72 to 8.00)	7.02	(3.25 to 13.02)

*Median age group, **within 30 days of admission
Adapted from Griffiths, Saville (4)

Table 4 Patient Day staffing characteristics for all days (n=626,313) and for days of low staffing

	All days			Days of low staffing		
**All**	**Mean**	**SD**	**Median**	**Mean**	**SD**	**Median**
**RN staffing**						
(% days below mean)	-			(45%)		
Hours per patient day	5.29	(4.22)	4.17	4.67	(3.88)	3.57
RN %	61%	(0.12)	61%	60%	(0.13)	60%
Senior RN (band 6+) %	25%	(0.18)	21%	26%	(0.20)	22%
Temporary Bank %	5.2%	(0.10)	0.0%	4.8%	(0.10)	0.0%
Temporary Agency %	4.9%	(0.10)	0.0%	4.1%	(0.10)	0.0%
**Assistant staffing**						
(% days below mean)	-			(45%)		
Hours per patient day	2.93	(1.37)	2.74	2.72	(1.19)	2.58
Senior NS (band 4) %	2.0%	(0.06)	0.0%	2.2%	(0.07)	0.0%
Temporary Bank %	12.3%	(0.19)	0.0%	11.2%	(0.19)	0.0%
Temporary Agency %	1.5%	(0.06)	0.0%	1.2%	(0.05)	0.0%

NS – Nursing support staff, RN - registered nurse staff, SD - standard deviation.
Adapted from Griffiths, Saville (4)

Univariable analysis results

Table 5: Exposures and outcomes for those exposed versus not exposed to understaffing

**Registered Nurse understaffing**		**Not exposed to understaffing**		**Exposed to understaffing**		**p**
Days	Mean(SD)	2.49	(1.45)	3.32	(1.40)	<0.001
Net low staffing (cumulative hours)	Mean(SD)	-3.36	(4.04)	1.15	(3.51)	<0.001
Length of stay	Mean(SD)	5.32	(10.67)	8.39	(14.43)	<0.001
Died	n (%)	4488	(4.0%)	27397	(5.3%)	<0.001
Readmitted	n (%)	15924	(14.3%)	78897	(15.3%)	<0.001
Total	N(%)	111414	(17.8%)	514899	(82.2%)	
**Nursing assistant understaffing**						
Days exposed	Mean(SD)	2.54	(1.47)	3.32	(1.40)	<0.001
Net low staffing (cumulative hours)	Mean(SD)	-3.05	(3.07)	0.88	(2.54)	<0.001
Length of stay	Mean(SD)	5.56	(11.50)	8.36	(14.31)	<0.001
Died	n (%)	4405	(3.9%)	27440	(5.4%)	<0.001
Readmitted	n (%)	16247	(14.2%)	78342	(15.4%)	<0.001
Total	N(%)	114363	(18.3%)	510517	(81.7%)	

Sensitivity analysis: Alternative understaffing thresholds

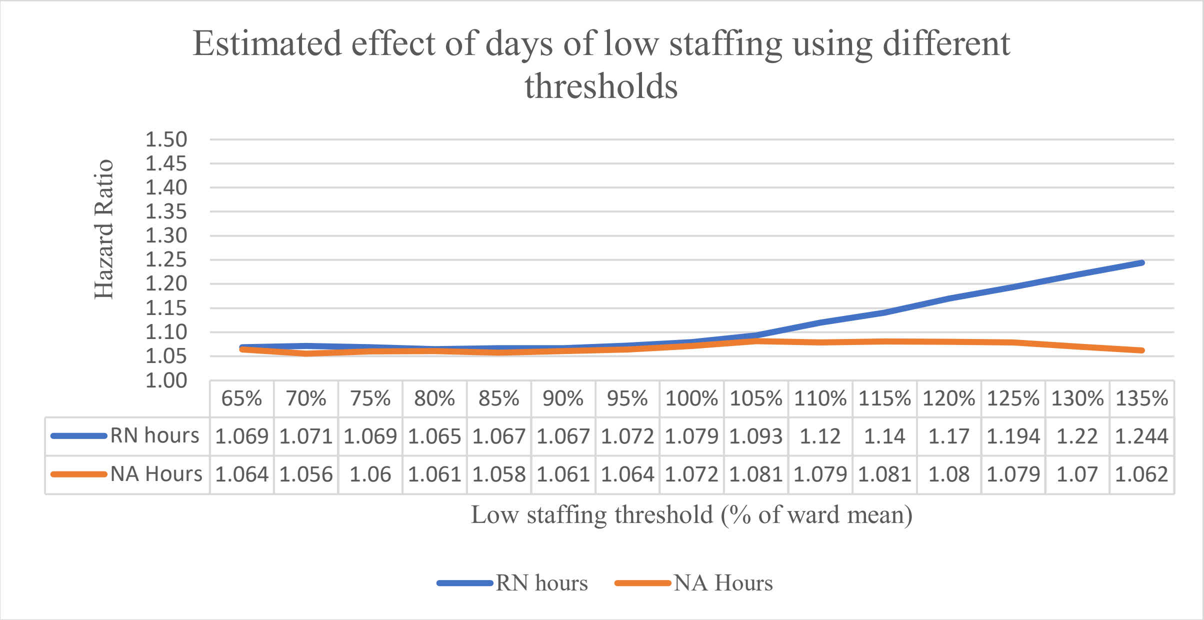

Figure 1 Hazard ratio for mortality for days of low staffing for different thresholds

References

1. Jones KC, Weatherly H, Birch S, Castelli A, Chalkley M, Dargan A, et al. Unit Costs of Health and Social Care 2022 Manual. University of Kent and University of York; 2022.

2. NHS England. National Cost Collection for the NHS. 2021/22 National Cost Collection data. 2023 [Available from: https://web.archive.org/web/20230818110747/https://www.england.nhs.uk/costing-in-the-nhs/national-cost-collection/.

3. NHS Digital. ISB 0070: Healthcare Resource Groups (HRGs) 2018 [Available from: https://web.archive.org/web/20230818122712/https://digital.nhs.uk/data-and-information/information-standards/information-standards-and-data-collections-including-extractions/publications-and-notifications/standards-and-collections/isb-0070-healthcare-resource-groups-hrgs.

4. Griffiths P, Saville C, Ball J, Culliford D, Jones J, Lambert F, et al. Nursing Team Composition and Mortality Following Acute Hospital Admission. JAMA Netw Open. 2024;7(8):e2428769.
